# The revival of thermal utilization from the Sun: interfacial solar vapor generation

**DOI:** 10.1093/nsr/nwz030

**Published:** 2019-03-04

**Authors:** Lin Zhou, Xiuqiang Li, George W Ni, Shining Zhu, Jia Zhu

**Affiliations:** 1 National Laboratory of Solid State Microstructures, College of Engineering and Applied Sciences, School of Physics, Key Laboratory of Intelligent Optical Sensing and Manipulation, Ministry of Education, Collaborative Innovation Center of Advanced Microstructures, Nanjing University, Nanjing 210093, China; 2 Department of Mechanical Engineering, Massachusetts Institute of Technology, Cambridge, MA 02139, USA

**Keywords:** solar vapor generation, solar absorber, solar evaporator, interfacial heating

## Abstract

Since solar energy is the ultimate energy resource and a significant amount of global energy utilization goes through heat, there have been persistent efforts for centuries to develop devices and systems for solar–thermal conversion. Most recently, interfacial solar vapor generation, as an emerging concept of solar–thermal conversion, has gained significant attention for its great potentials in various fields such as desalination, sterilization, catalysis, etc. With the advances of rationally designed materials and structures and photon and thermal management at the nanoscale, interfacial solar vapor generation has demonstrated both thermodynamic and kinetical advantages over conventional strategies. In this review, we aim to illustrate the definition, mechanism and figures of merit of interfacial solar vapor generation, and to summarize the development progress of relevant materials and applications, as well as to provide a prospective view of the future.

## INTRODUCTION

Solar energy, the most promising renewable energy source available on the surface of the Earth, enjoys an annual abundance of 3400 000 EJ, which is 10 times larger than the total estimated non-renewable energy resources. Just 0.1% of the total solar resource would be sufficient to meet the annual worldwide demand for energy [1]. Accordingly, the development of solar–thermal energy conversion has a long history, ranging from solar water heaters, solar cookers, solar driers, solar ponds, to solar desalination and solar power plants [2], etc. Among various forms, solar vapor generation, which particularly refers to solar steam under 100°C, has recently been studied for clean water generation, domestic sterilization, and electricity generation [3–5], and is particularly desirable for off-grid areas [6]. However, the efficiency of solar vapor generation has been very low (∼24% with high optical concentration [3]) until recently. Attributable to recent advancements in photon and thermal management and materials and structural design at the nanoscale, interfacial solar vapor generation (ISVG) has emerged as a novel concept for solar–thermal conversion. Like the early stage of each solar conversion device, standards for ISVG’s figures of merit (FOMs) and performance measurements are still nascent, but maturation is critical for sustainable development. In this review, we aim to provide a fundamental understanding of ISVG thermodynamically and kinetically, to define the FOMs and build measurement protocols, and to summarize the state of the art in the field as well as provide future opportunities. There are a couple of recent reviews covering detailed discussions on materials composition [7], structural design [8], efficiency comparison and concrete applications [9,10], etc., which will not be discussed in this review.

**Figure 1.**
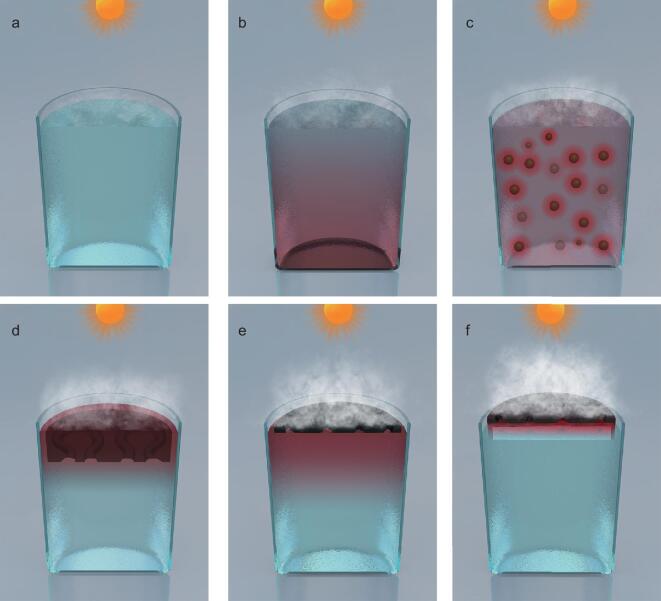
Development pathways of interfacial vapor generation driven by solar irradiance. (a) Natural evaporation of bulk water. (b) Conventional solar pond evaporation with black coating at the bottom. (c) Suspended nanoparticles (absorber) enabled evaporation. (d) Suspended absorber (below the water/air interface) enabled evaporation. (e) Direct-contact absorber enabled evaporation at the water/air interface. (f) Indirect-contact absorber enabled evaporation.

Figure 1 illustrates the development pathways of the advanced solar vapor generation. Natural water evaporation (Fig. 1a), such as that from oceans, rivers, lakes, etc., forms the basis of the global hydrologic cycle. However, due to poor light absorption in water, natural solar evaporation is inefficient (∼<20% of solar energy transferred estimated by reported literature [11]). Ancient technologies, such as solar stills or solar ponds, increased light absorption from the Sun by painting the bottom of the water reservoir black (Fig. 1b). However, as most of the harvested solar energy is absorbed at the bottom of bulk water, far from the evaporation sites, the absorbed solar energy is mainly used to heat the bulk water, rather than to directly facilitate vapor generation. Therefore, the overall solar evaporation efficiency is still low (∼<40%) [12], unsuitable for point-of-use applications.

Recently, significant efforts have been made to increase the relatively low energy transfer efficiency of conventional solar vapor processes by leveraging recent developments in nanotechnology [3,5,13,14]. By immersing metallic [3,15], carbon [16] or other nanoparticles [17,18] in the bulk water (Fig. 1c), a so-called optonanofluid [19,20] is formed and the solar-to-vapor-generation can be expected to increase due to enhanced light absorption and decreased heat resistance. It was soon recognized that volume of the locally heated water should be further reduced to minimize unnecessary heating toward the bulk water. In 2014, Hogan *et al.* proposed greatly increasing the concentration of gold nanoparticles (Fig. 1c), to optically confine heating to the water–air interface [21]. Another approach thermally confined heating: Ghasemi *et al.* demonstrated a carbon-based suspended foam structure [11] (Fig. 1d), which used porous insulating foams to prevent generated heat from escaping to the bulk water. This structure formed a prototype of ISVG, and subsequently a variety of self-floating solar evaporators [13,22–25] extended this concept by squeezing the heating volume in much thinner layers (Fig. 1e). Most recently, Li *et al*. [14] (Fig. 1f) proposed a unique interfacial solar vapor generation design by using bottom thermal insulators combined with confined water paths. Because of the efficient decoupling of the evaporated water area with bulk water in the spatial scale, such ISVG for the first time increases the thermal responsivity and the solar vapor efficiency up to 80% without extra optical concentration or container insulation. Most importantly, the pronounced solar vapor efficiency is independent of the water volume, a critical signature of ISVG. This current form of ISVG advantageously enables direct deployment into existing bodies of water with minimal preparation. However, without loss of generality, the cases in Fig. 1c–e have also been subscribed to the field of ISVG as in most of the literature [26–29].

## PHYSICAL PICTURE AND FIGURES-OF-MERIT OF INTERFACIAL SOLAR EVAPORATION

### Definition and physical picture of ISVG

**Figure 2.**
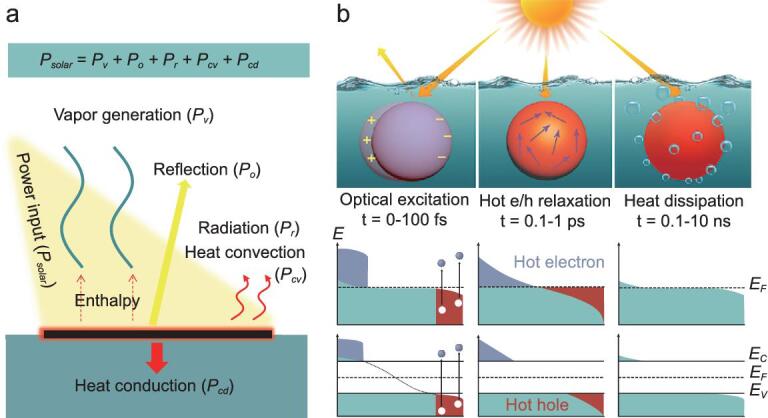
Schematic (a) macroscopic and microscopic physical pictures (b) of the solar-to-thermal conversion process for solar vapor generation. Suppose that the sphere in (b) represents the elementary building block of the solar evaporator in (a).

In definition, an ideal ISVG (Fig. 2a) refers to a liquid–gas phase-change process of water driven by solar energy in close proximity to the bulk water surface. In order to reveal the unique advantages of interfacial solar heating, microscopic and macroscopic pictures of the ISVG are sketched.

In the microscopic view of carrier dynamics, without loss of generality, the time-dependent ISVG process of a representative building block or meta-atom (e.g. a nanosphere, used here for simplicity) of the solar evaporator is illustrated in Fig. 2b. It can be divided into three sub-processes (upper sub-figures) [30], with the lower sub-figures respectively depicting the typical carrier population profiles for metallic and semiconductor materials. Note that the highly absorptive organic solar nanomaterials are quite similar to inorganic semiconductors by making an analogy of the lowest unoccupied molecule orbit (LUMO) to conduction band (*E*_c_ in Fig. 2b) or the highest occupied molecule orbit (HOMO) to valance band (*E*_v_ in Fig. 2b) respectively, which are ascribed to generalized semiconductors in this review without separate discussions. Other dielectric materials with extremely large bandgaps (e.g. *E*_g_ = *E*_c_ - *E*_v_ >4 eV) are almost transparent in the solar spectrum, which cannot serve as primary absorptive constituents but host media. Taking the metallic nanoparticle as an example, the first step of ISVG is the optical excitation of hot carriers. Sunlight is incident on the nanoparticle, inducing optical excitations and electron oscillations (or carrier transitions) in nanoparticles, which effectively absorb the input solar energy on a timescale of less than 100 fs. The probability of photon absorption and carrier transition is proportional to the local absorption power inside the particle, which depends on the material type. In the second step, absorbed solar energy is converted to kinetic energy and thermally relaxed via Landau damping, electron–electron scattering and electron–lattice scattering processes inside the nanoparticle on a timescale of 0.1 - 1 ps, leading to hot carrier relaxation inside the nanoparticle as lattice vibration. Finally, the photothermal energy is dissipated to water molecules. Vibrational coupling between lattices of particles (primarily in the form of low-frequency acoustic phonons) and molecules of water assists the energy transfer to the water–vapor transition enthalpy. Note that different materials possess quite different characteristic timescales in this step. For example, for noble metals or semiconductors of lower interfacial thermal resistance, it is around 100 ps while for other materials of higher thermal resistance, it would be longer (∼10 ns or more) [30]. A steady-state intense solar vaporization can be attained up to 1 s or even longer based on reports in the literature so far; this depends on the material types, particle sizes, and thermal properties of the surrounding water as well the solar irradiance power density. On condition that the building blocks are wrapped with extremely small volumes of water or ultrathin water film (the extreme microscopic case of ISVG), ISVG would have a much faster response than bulk solar evaporation due to reduced thermal mass.

From the macroscopic point of view, when the meta-atoms (sphere in Fig. 2b) build up and work as an ideal ISVG evaporator (Fig. 2a), the converted solar heat is well localized to a minimal volume of water near the interface [2,26,27]. Therefore, an ideal ISVG can directly utilize the input solar power *P* for vapor formation without heating the bulk water underneath, beneficial for strongly minimizing thermal conduction loss *P*_cd_ (most remarkable dissipation channel) and thus being more directionally transferred to the enthalpy of the liquid–vapor transition (*P*_v_). Due to these thermodynamic advantages of heat localization and the increased heat resistance at the interface between air and the underlying bulk water originating from the insulating material, an ideal ISVG can enable a higher vapor production rate and energy utilization efficiency, a higher vapor temperature and a shorter response time compared to conventional bulk water evaporation systems, as depicted in Fig. 1a and b.

### Figures of merit of ISVG

During this early stage of ISVG development, it is crucial to standardize the FOMs and build comparable protocols of the ISVG process to lay a solid foundation for the sustainable development in this fast-evolving field. Based on the above thermodynamic analysis, we propose four key parameters or FOMs for evaluating different solar vapor systems, namely, vapor production (evaporation rate), vapor temperature, energy transfer efficiency (directionally utilized for vapor generation) as well as response time. These intrinsic parameters that characterize the performance of solar vapor generation can be affected by extrinsic parameters such as the initial states (e.g. initial water temperature) and/or boundary conditions (e.g. environmental temperature).

#### Vapor production rate

Solar vapor production is one of the most important parameters for ISVG, especially for applications requiring large volumetric vapor outputs, such as seawater desalination, wastewater treatment and so on. The production rate of solar vapor can be evaluated by the solar-driven steady-state evaporation rate per unit area ($\dot{m}/A$). Generally speaking, higher light intensity leads to a higher rate of vapor generation. However, it strongly depends on various extrinsic parameters, such as humidity, ambient temperature, thermal insulation conditions, wind speed, etc.

#### Vapor temperature

Vapor, the gaseous phase of water, can carry energy as it flows. In thermodynamics, vapor or steam quality is directly related to its temperature and pressure. For solar evaporation, here the vapor temperature largely determines its potential applications. For example, solar steam with a high temperature (>500°C) is advantageous for steam-driven engines for electricity generation, while solar steam of 120–150°C can be ideal for off-grid or domestic medical sterilization. Even for solar vapor with *T*_v_ ∼<100°C, the utilization processes can be quite different. Solar vapor with *T*_v_ >110°C can be employed for multi-stage evaporation (MSE). Solar vapor with *T*_v_ >70°C can be used for multiple effect distillation (MED). Solar vapor with an even lower temperature can be directly used for thermal-based distillation (*T*_v_ ∼<50°C) [31] or membrane desalination (*T*_v_ ∼<80°C) [32,33], respectively.

#### Energy transfer efficiency

To quantify the energy conversion capability, the energy transfer process of an ISVG can be divided into two cascaded processes; one is solar energy harvesting and thermalization, the other is directional transfer of the thermalized energy to phase-changing vaporization. Supposing that all of the absorbed solar power can be completely thermalized via a non-radiative decay process (existing in most reported photothermal materials), the extrinsic and intrinsic solar–thermal efficiency of ISVG can be defined as in Equation (1) by making an analogy to the quantum efficiency of a PV cell [34]:

(1) \begin{equation*}{\eta ^{{\rm{ex}}}} = \frac{{A{P_{{\rm{abs}}}}}}{{A{P_{{\rm{solar}}}}}},\,{\eta ^{{\rm{in}}}} = \frac{{\dot{m}{h_{{\rm{LV}}}}}}{{A{P_{{\rm{abs}}}}}}.\end{equation*}

The extrinsic efficiency *η*^ex^ represents the overall conversion efficiency from input solar power *P*_solar_ to heat, determined by the solar absorptance *α*_eff_. Without specifying the non-radiative decay channels of the absorbed solar energy, *η*^ex^ has also been employed to evaluate the solar-to-thermal conversion systems in the dry state and provide insights into the photothermal response and thermal capacities [35]. In contrast, the intrinsic efficiency *η*^in^ aims at revealing to what extent the absorbed solar power *AP*_abs_ can be directionally transferred to the phase-change enthalpy of water. $\dot{m}$ refers to the mass evaporation rate, and *h*_LV_ is the phase-change enthalpy of water, including both sensible heat and latent heat during the steady-state solar vapor generation process.

The combination of the above two expressions gives rise to the definition of solar-to-vapor conversion efficiency:

(2) \begin{equation*}{\eta _{{\rm{solar}} - {\rm{steam}}}} = \frac{{\dot{m}{h_{{\rm{LV}}}}(T)}}{{A{P_{{\rm{solar}}}}}}.\end{equation*}

To identify the net solar-driven evaporation efficiency, the contribution of the dark evaporation should be considered carefully. If the surface temperature of solar evaporators *T*_s_ is lower than the ambient temperature *T*_amb_ [36–38,39], Equation (2) can be expressed as

(3) \begin{equation*}{\eta _{{\rm{solar}} - {\rm{steam}}}} = \frac{{{{\left. {\dot{m}{h_{{\rm{LV}}}}} \right|}_{{\rm{solar}}}} - {{\left. {\dot{m}{h_{{\rm{LV}}}}} \right|}_{{\rm{dark}}}}}}{{A{P_{{\rm{solar}}}}}}.\end{equation*}

Here ${\dot{m}_{{\rm{solar}}}},{\dot{m}_{{\rm{dark}}}}$ are the evaporation rate of ISVG under solar illumination and in the dark, respectively. For a solar evaporator with surface temperature (*T*_s_) higher than the ambient temperature (*T*_amb_), as the evaporation will not benefit from ambient, the influence of dark evaporation should be neglected.

#### Response time

Response time is defined as the rise (drop) time of an ISVG system that requires establishment of the thermodynamic steady state (with stationary evaporation rate and vapor temperature). Thanks to the inherent heat localization effect, the response time of ISVG is typically faster than bulk evaporation systems. In addition, it is dependent on the relative thermal mass of the insulating materials, surroundings as well as interfaces. For example, due to much higher density of free electrons and energy states above the Fermi energy level *E*_F_ as well as higher thermal conductivity, metallic materials can respond faster than semiconductors in almost all three microscopic processes of Fig. 2b.

It is worth noting that, although all of the above four parameters are crucial for ISVG systems, it is rather hard to maximize all the parameters at the same time since they are inter-correlated. For example, for a fixed solar input power, a higher vapor temperature typically means a larger thermal energy loss and thus a lower energy transfer efficiency, and a higher rate evaporation at one sun is commonly accompanied by a low vapor temperature. Evaporation rate and energy transfer efficiency are another coupled pair with a far from linear-dependent relationship [40]. Therefore, for different applications, the primary FOMs can be defined differently. For example, for the purpose of generation of water purification, people would like to employ vapor production rate as the primary FOM for system evaluation, while for solar sterilization for medical purposes, the solar vapor temperature would be more crucial [4]. The latest developments in ISVG show that the environment can contribute significant amounts of energy, compared with the solar contribution [36–38]. Thus, the absolute evaporation rate can be directly used without subtracting the dark evaporation for evaluation under these circumstances. Depending on the particular applications, there is a series of auxiliary issues on the materials, structures or systems that need to be evaluated, such as the raw material cost, scalability, flexibility, long-term stability, etc. Some of these issues may dominate the overall evaluation. However, the four main FOMs laid out here are still generally the most important metrics to assess ISVG performance.

### Protocols of ISVG

Except for the FOM parameters, a second most important issue is how to build protocols for measuring these FOMs. Actually, solar vapor generation is a thermodynamic process that is far from a quasi-static process. The representative FOMs such as efficiency, evaporation rate and vapor temperature depend on the initial and boundary conditions. The detailed experimental setup for ISVG measurements is summarized in Supplemental Note S1, in which the real-time experimental setup, optical/thermal calibration and FOM measurements are included. A few important notes are listed below:

- A well defined solar-to-vapor efficiency must refer to the exact thermodynamic steady state, which requires not only the invariant evaporation rate but also the steady temperature profile of the absorber and vapor.
- The evaporation rate $\dot{m}$ should be the vapor production rate produced by the solar energy. Therefore, it is worth emphasizing again that the dark evaporation or vapor generated by the surroundings should be carefully treated; this is highly dependent on the operation temperature as discussed in the Section ‘Figures of merit of ISVG’.
- The expression in Equation (3) is obtained with the hypothesis of spatial uniformity for both solar irradiance and vapor generation. If the absorber or light source is not uniform, the power in Equation (3) should be replaced with spatially integrating forms. Therefore, it is optimal to add a reflecting aperture and the total power passing through aperture should be measured (Supplemental Fig. 1a).
- When the efficiency is obtained, the energy balance can be evaluated from the thermodynamic perspective (equation in Fig. 2a). Note that such methodology can only provide a general estimate of the solar vapor efficiency provided that the steady state arrives and all the traditional thermodynamic parameters can work.
- The comparison of solar vapor efficiency is really difficult. One should try to offset a lot of external boundary conditions that may introduce any potential prejudice. For example, the ambient temperature and humidity are vital for water evaporation; however, they vary distinctly for different areas of the world. Another important parameter is the size of the evaporation area. A small area will evaporate proportionally faster than a large area, due to factors like confinement of vapor flow direction to 1D, faster replenishment of dry air in a small system, etc. Therefore, it is unfair to compare a 1cm^2^ system with a 100 cm^2^ system. The operating temperature will decrease simply from reduced size, even assuming perfect side insulation. Water quantity may be another trick parameter especially for the non-ideal ISVG systems. We recommend that all researchers report these external parameters clearly so that readers can evaluate the reported data and/or indicated results more effectively.
- ISVG is a coupled process of energy conversion and mass transfer. Water supply channels for continuous vapor generation act in an opposite way to thermal insulation. For example, once a water path is established, water is pumped towards the evaporation area. However, the heat is also conducted along the same path. The trade-off exists in various ISVG systems. Therefore, optimization may simultaneously involve both energy and mass transfer.

## RATIONAL DESIGN FOR INTERFACIAL SOLAR VAPOR GENERATION

The past few years have witnessed the developments of ISVG with various rational designs for the three key components outlined in Fig. 2, including manipulations on the optical absorption and thermal transfer dissipation as well as mass transfer such as water pumping and vapor escape. Herein we will give an overview of the basic frameworks and summarize recent progresses on these three key components.

### Photonic manipulation of solar evaporators

ISVG is first of all a photo-physics process. Efficient light harvesting is crucial for maximizing the external quantum efficiency *η*^ex^ of ISVG, a necessary condition for any highly efficient solar-to-thermal conversion system. Light absorption is one of the most elementary optical properties of materials, which is a signature of light–matter interactions, or interactions between photons irradiated by the Sun and electrons and/or phonons in solar absorbers. Absorption bandwidth and absorption efficiency are two crucial issues for optical designs on solar absorbers of ISVG. For weak dispersive solar absorbers, one can employ the average efficiency to qualitatively evaluate the absorption performance without special considerations of the bandwidth. To quantitatively evaluate the overall absorption performance of various solar absorbers, the solar absorptance *α*_eff_ can be employed, which reads as [41]

(4) \begin{equation*}{\alpha _{{\rm{eff}}}} = \frac{{\int{{A(\lambda ){I_{{\rm{solar}}}}(\lambda )d\lambda }}}}{{\int{{{I_{{\rm{solar}}}}(\lambda )d\lambda }}}},\end{equation*}

where *I*_solar_(*λ*) is the solar spectral irradiance referring to the hemispherical irradiated power per unit area per unit wavelength from the Sun, 99% of which is in the wavelength range of 280–2500 nm. *A*(*λ*) in Equation (4) represents the absorption efficiency, which is measured across all the 2π solid angle of the diffraction space. Due to the inherent spectral characteristics of solar irradiance and the peculiar operation condition of ISVG, solar absorbers with broad absorption bandwidths, wide angular tolerance and polarization insensitivity, as well as humidity and chemical stability, are highly desirable. Basically, by careful choice of the photothermal materials and designing the structures rationally and accordingly, micro/nanostructured solar absorbers can enable maximal solar absorption with minimal optical loss.

Two primary categories of photothermal materials are metals and generalized semiconductors (including organic semiconductors and carbon-based materials). Due to extremely different dielectric responses and absorption mechanisms, the optical designs of broadband absorption for the two types of materials are very different; they are mainly determined by the electronic structures of nanomaterials (intrinsic strategy) and the photonic band of nano/microstructures (extrinsic strategy), respectively.

#### Photonic manipulations for metal-based solar absorbers

Metals possess huge numbers of free electrons (on the order of 10^22^–10^23^ cm^−3^). The mechanism of light absorption in metals is related to the absorption by these free electrons in the conduction band, such as Landau damping, electron–electron scattering, electron–photon scattering, photon–photon scattering, etc. Because of the extremely high carrier concentration, the absorption coefficients of metals can reach up to 10^−6^ cm^−1^ in the solar spectrum, making them good candidates for photothermal materials. On the other hand, the unique negative dielectric response of metals makes them unsuitable for bulk mode propagation (highly reflective) and means that they only support surface plasmon modes. Surface plasmon modes are coupled optical modes of collective oscillations of free electrons near metallic surfaces, originating from the long-range Coulombic interaction and inertia of the surface free electrons. The periodic metallic surfaces can support surface plasmon polariton (SPP) modes while metallic nanoparticles with curved surfaces possess localized surface plasmon (LSP) modes. Because of the unique capability of sub-wavelength light confinement near metallic surfaces, LSP-induced morphology-tunable light absorption, as well as scalable fabrication processes, metallic nanoparticles have received considerable interest for ISVG.

**Figure 3.**
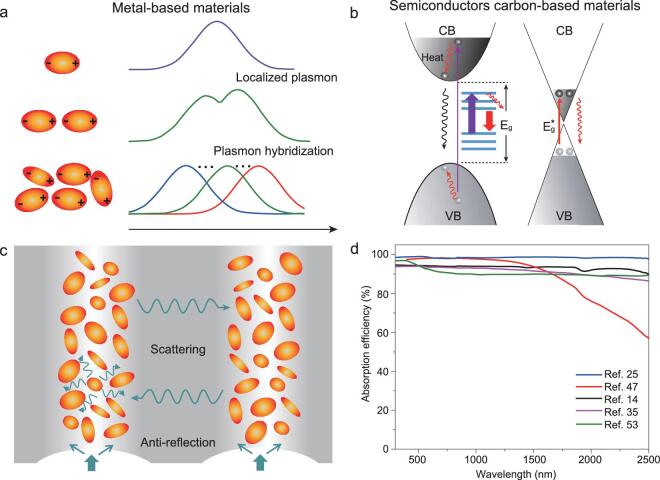
Optical properties and nanophotonic designs for solar absorbers of ISVG. (a, b) Mechanisms of broadband absorption for (a) metal-based and (b) semiconductor-based, as well as carbon-based solar absorbers, respectively. (c) Ideal nanophotonic design for highly efficient and broadband solar absorber of ISVG, which is replotted based on [25]. (d) Measured light absorption performance of several representative solar absorbers of ISVG. Data are extracted and/or recalculated based on [25,47,14,35,53] as shown in the legend.

The key issue for plasmonic solar absorbers is how to enable the broadband absorption. The mechanism for broadband absorptive metals is mainly based on artificial engineering of photonic band structures [42–44], which is enabled by nano/microstructure design of metallic nanoparticles and thus called an extrinsic strategy (Fig. 3a) [45]. In pioneering work, Halas group reported gold/SiO_2_ nanoparticle fluid for solar vaporization [3], which inspired the revivals of the interfacial solar evaporation field. A self-floating gold film paper with a light absorption of 85% in the range of 400–800 nm was then reported [13]. Most recently, Zhu group demonstrated ultra-broadband gold-based plasmonic absorbers with 3D gold nanoparticle porous structures. By fine-tuning the structure and deposition conditions, an average measured absorbance of 99% across a wavelength range of 400 nm–10 μm was demonstrated [25], shedding light on ideal optical designs for solar absorbers. The schematic mechanism is shown in Fig. 3c, which depicts three key components for an ideal optical absorber of ISVG: highly porous structures (extremely low effective refractive index *n*, with pore sizes comparable to the wavelengths for strong scattering enhancement), highly absorptive materials (with high extinction coefficient *κ*) and multiple meta-atoms for a high density of optical modes. So far, various metallic structures have been reported for solar–thermal conversions, such as nanoparticles [25,46,47], nanowires [24], core–shell or nanosheet mesoporous structures [48–50]. Apart from gold [3,13,15,24,25,46], similar photonic designs have been reported in other plasmonic material systems, such as plasmonic wood [46], aluminum [47], silver [51,52], indium [49], Cu_2_ZnSnS_4_ [50], etc. Through the smart nanophotonic designs, the maximal absorption bandwidth of plasmonic absorbers can be flexibly tuned, with a maximal bandwidth comparable to the carbon-based absorbers [24,25].

#### Photonic manipulations for semiconductor-based solar absorbers

The absorption mechanisms of semiconductor-based materials are more complicated than metals. In semiconductors in low-temperature-operated ISVG systems, there is only a small concentration of free electrons, and light absorption is determined mainly by bound electrons (for intrinsic semiconductors), which can be achieved by interband electron transition for above-bandgap photoexcitation (*hν* > *E*_g_). Absorption below the bandgap *E*_g_ of semiconductors is commonly related to excitons, electronic transitions between impurity levels, and free carrier absorption as well as lattice-vibration-induced absorption, etc. In order to satisfy the broadband solar absorption requirement, there are a couple of rational designs on materials and/or microstructures. One of the desirable semiconductor-based solar absorbers is the narrow-bandgap intrinsic semiconductors (the critical bandgap *E*_gc_ < ∼0.5 eV refers to the absorption edge of 2500 nm). As the photon energy is higher than the bandgap, a large number of electron–hole pairs with higher energy will be generated, relax to the band edges and recombine at a much lower energy near the band edge, resulting in much higher light-to-heat conversion efficiency compared to wide bandgap semiconductors. Apart from intrinsic narrowband semiconductors, semiconductors with a high density of doping energy levels are feasible alternatives as well. Figure 3b illustrates the two absorption mechanisms of these semiconductor materials, in which black arrows stand for the optical transition and red arrows for the thermal relaxation respectively. Intuitively, both the thermal relaxation and non-radiative recombination are ideal for efficient photothermal materials. Recently, a number of cost-effective and non-toxic semiconductors have encountered explosive developments for solar evaporators, such Ti_2_O_3_ [53], hydrogenated black titania [54], Ti_3_C_2_ [55], Fe_3_O_4_ nanoparticles [56], oxygen-deficient MoO_3_ quantum dots [57], and BiInSe_3_ [48] as well as some bimetal oxides [58] as standalone solar evaporators. In the meantime, a variety of nano/microstructures such as nanospheres [46,53,56], nanosheets [55], and 3D structures [37] have been developed as well, the structural designs of which resemble the basic components in the structural designs of metallic solar absorbers, such as multiple scattering enhancement and effective reduction in effective refractive index. Very recently, by fully utilizing the intrinsic material properties as well as geometric resonances, Yang group further reported a novel Te nanoparticle-based photothermal material, in which multiple optical mode designs are employed for broadband absorption [59].

Among the two representative photothermal materials discussed above, the most popular candidate for ISVG is carbon-based materials (right panel in Fig. 3b). Basically, carbon-based materials possess the advantages of both metals and semiconductors. In carbon-based materials, there are high densities of 2D free electrons (also called weakly held electrons) due to the peculiar lamellar structure. Under light illumination with small energy input, these electrons can be easily excited from the π orbital to the π* orbital, and can then be excited from the ground state (HOMO) to a higher energy orbital (LUMO). Once the photon energy matches these electronic transitions well, these excited electrons will vibrate strongly and transfer the solar energy via heat when these excited electrons relax back to their ground state. Such semiconductor-like band structures make carbon-based materials much more suitable for broadband solar absorption provided that the thickness is sufficient (usually mm–cm), which greatly reduces the limitations of structural designs like metallic absorbers. Therefore, plenty of complicated double-layer evaporator designs with well defined thermal and water path control exist compared to metal-based ISVG (as demonstrated below). So far there are a couple of transformative sub-materials employed for ISVG systems, such as graphite [11], graphene [22,35,40,60], graphene oxide (GO) [14,61–63], reduced graphene oxide (rGO) [64–66], carbon nanotubes (CNTs) [67,68], graphdiyne [69], carbon black (CB) [70,71], etc. Very recently, carbon-based photothermal materials have been further extended into various carbonized organic materials including PPy [22], natural woods [46,56,72–74] and polydopamine [75], and biomass [76]. Apart from versatile material designs, similar nanophotonic microstructure designs suggested in metal-based systems have been employed in carbon-based materials as well. For example, Liu group developed a hierarchical graphene foam with graphene flakes assembled in a 3D framework, resulting in >90% solar–thermal conversion efficiency [35]. One can refer to [27] for a more detailed review of carbon-based absorbers for solar vapor generation.

Figure 3d shows the measured absorption spectra of several representative solar absorbers for ISVG. Due to rational nanophotonic design, both semiconductor-based and metal-based absorbers can enable highly efficient, broadband solar absorption across the entire solar spectral range. Most of the reported structures have a high effective absorptivity of *α*_eff_ >95%. However, unlike intrinsic absorptive materials, metal-based solar absorbers can be more flexible for simultaneously steering absorption bandwidth and intensity. For example, metallic structures can be employed for spectrum selective solar absorbers [77,78], which can manipulate the absorption properties in different wavelength regimes flexibly, beneficial for thermal emission management in concentrated solar energy applications [79]. It is worth noting that up to now most of the photonic designs for solar evaporators have been based on optical absorptance of dry states of samples. More precise evaluation on both optical absorption and thermal emissivity requires that wet solar evaporators with similar water filling conditions are considered.

### Thermal management of solar evaporators

**Figure 4.**
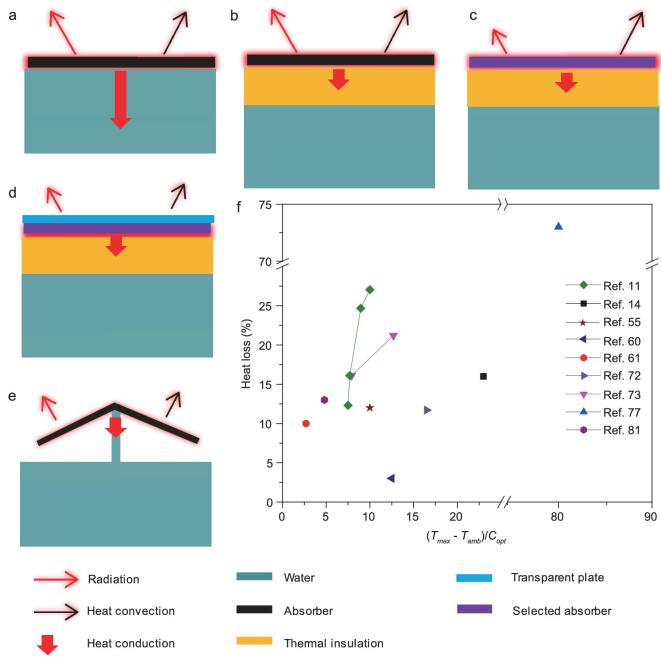
Thermal management for ISVG. (a) The species of heat loss of ISVG. (b) Schematic of ISVG with suppression of conduction loss. (c) Schematic of ISVG with suppression of conduction loss and radiation loss. (d) Schematic of ISVG with suppression of conduction loss, convection loss and radiation loss. (e) Schematic of ISVG with suppression of conduction loss, convection loss and radiation loss through reducing the evaporation temperature. (f) Comparison of heat loss as a function of (*T*_max_ - *T*_amb_)/*C*_opt_ among different interfacial evaporation systems. *C*_opt_ is defined as the optical concentration factor of the input concentrated power normalized by the irradiated solar power of one sun. Data are extracted and/or recalculated based on [11,14,55,60,61,72,73,77,81] as shown in the legend.

Proper nanophotonic designs can greatly promote the light-harvesting capability in ISVG systems by suppressing optical losses; however, early-stage ISVG systems (such as the suspended and some self-floating systems) still suffered from low overall energy transfer efficiency, especially at one sun illumination. This is due to absorbed energy being severely wasted in three primary channels of unwanted heat losses: conduction, radiation, and convection (Fig. 4a). For example, it is estimated that there is about 7% radiation loss, 5% convection loss and 43% conduction loss, if the absorber is in direct contact with bulk water without any thermal management design [61], as shown in Fig. 4a. The heat flux per unit area *q* caused by the conduction, radiation and convection losses are expressed by the Stefan–Boltzmann law, Fourier's law, and Newton's law of cooling, respectively:

(5) \begin{eqnarray*} {q_{{\rm{cond}}}} &=& {\rm{ - }}{\kappa _{\rm{T}}}\nabla T, \nonumber\\ {q_{{\rm{rad}}}} &=& {\sigma _{{\rm{SB}}}}({\varepsilon _{{\rm{eff}}}}T_{{\rm{abs}}}^4 - {\varepsilon _{{\rm{amb}}}}T_{{\rm{amb}}}^4),\nonumber\\ \quad {q_{{\rm{conv}}}} &=& {\rm{h(}}{T_{{\rm{abs}}}} - {T_{{\rm{amb}}}}), \end{eqnarray*}

where *σ*_SB_ is the Stefan–Boltzmann constant and *T*_abs_ and *T*_amb_ are the temperature of absorber and the ambient, respectively. All of these thermal loss mechanisms are dependent on both the intrinsic thermal properties of the absorbers (thermal emissivity *ϵ*_eff_ and thermal conductivity *κ*_T_) and the extrinsic thermal conditions (temperature difference, gradient or heat convection coefficient *h*), which can be rationally designed from the absorber and system levels, respectively. The aim of thermal management is to suppress these heat losses by using advanced materials and/or system designs (referring to intrinsic and extrinsic strategies, respectively).

#### Suppressing thermal conduction loss

Thermal conduction dissipation to bulk water is the most serious loss channel among the various thermal dissipation mechanisms. Up to now, management strategies of suppressed thermal conduction loss have included intrinsic and extrinsic ones, aiming to decrease the two items *κ*_Τ_ and *dT/dx* respectively.

##### The material design.

The material design (intrinsic strategy) is to reduce thermal conductivity *κ*_Τ_ through microstructural design of nanomaterials. A promising candidate is microporous structures. In microporous structures, the framework of pore walls has relatively high intrinsic thermal conductivity (compared to air), while the high volume ratio of air pores lowers the overall effective thermal conductivity. In the past few years, for heat localization and to reduce conduction losses, some microporous absorbers (nanofiber aerogel [80], graphite/carbon foam [11], wood [73], paper-based AuNP [13] and reduced graphene oxide/nanocellulose [81]) have been employed for ISVG, with claimed thermal conductivities of ∼0.06–1 W/mK. In most designs, the microporous absorbers were in direct contact with bulk water, and interconnected pores allowed water to infiltrate and supply the evaporation processes and also allowed vapor to escape. However, water in the micropores can severely degrade the thermal insulation, and most reported thermal conductivities were measured in the dry state instead of the wet state. In many cases, wet microporous structures could have much higher thermal conductivities than in the dry state. Effective demonstration of low thermal conductivity materials for ISVG should consider wet materials similar to those in operating conditions. Properly designed microporous structures should optimize the amount of water infiltrating the pores to balance an adequate water supply, while maintaining minimal thermal conductivity. However, microporosity optimization remains highly challenging, and so far most reports of high solar-to-vapor conversion efficiency (∼80%) have been achieved with costly insulating containers or optical concentration [11,25,47].

Reducing thermal conduction losses also requires proper geometric design of the thermal insulating materials. For example, thin layers of low thermal conductivity materials can still result in high thermal conductance, the extrinsic measure of a system's propensity to conduct heat. Solar evaporators with rather low thermal conductivity but extremely small thickness will be harmful to conduction loss as well. Proper reduction of thermal conduction losses requires more attention to heat flux instead of only thermal conductivity. Meanwhile, the design of permeability of material is also important during suppression of heat loss with a confined water path. In general, the principle of design of permeability is that the water supply rate should be larger than the real evaporation rate to guarantee an effective water supply. Otherwise, the conversion efficiency will be influenced by the water supply. For example, Hu *et al*. demonstrated that the solar-to-vapor conversion efficiency can increase from 56% to 77% just by increasing the permeability of reduced graphene oxide by adding biocompatible sodium alginate [64].

##### The system/structure design.

The system/structure design (extrinsic strategy) on suppressing conduction loss is to constrain heat transfer with smart design of the thermal insulating structure. One approach is to physically separate the evaporator from the bulk water. As shown in Fig. 4b, a floating polystyrene-foam-based solar evaporator was reported by Li *et al.* in 2016 [14], with GO serving as the solar absorber material. Since polystyrene foam is very thermally insulating (*κ* ∼0.04 W/mK) and hydrophobic, this ISVG concept can simultaneously reduce the effective thermal conductivity of the overall system as well as the temperature gradient, localizing the heat to a thin water layer. Therefore, it effectively resolved the aforementioned paradox between water supply and suppressed conduction loss, and achieved a 78% solar-to-vapor conversion efficiency without insulating containers or optical concentration. Only 5% of the total input solar energy was estimated to be wasted through conduction losses. Furthermore, because of the minimized conduction loss, the energy transfer efficiency is independent of the volume of water being evaporated, which is beneficial for large-scale applications. Currently, this strategy is widely used, and higher efficiency (∼83%) can be obtained through system optimization [82].

#### Suppressing thermal radiation loss

Radiation loss to the ambient is another crucial energy loss channel, especially for high-temperature ISVG systems. As depicted in Equation (5), thermal radiation loss in ISVG systems is mainly due to higher evaporator temperatures compared to the ambient, resulting in a net flux of infrared photons to the ambient. Suppression of thermal radiation can be achieved by both intrinsic and extrinsic designs, to reduce the effective thermal emissivity *ϵ*_eff_ by employing selective absorbers (Fig. 4c), or to decrease the surface temperature *T*_abs_ of the solar evaporator.

##### The intrinsic strategy to decrease radiation loss.

The intrinsic strategy is based on spectral engineering of infrared thermal emission of the structured materials, which is directly characterized by *ϵ*_eff_ via the formula below:

(6) \begin{equation*}{\varepsilon _{{\rm{eff}}}}({T_{{\rm{abs}}}}) = \frac{{\int{{A(\lambda ){I_{{\rm{BB}}}}(\lambda ,{T_{{\rm{abs}}}})d\lambda }}}}{{\int{{{I_{{\rm{BB}}}}(\lambda ,{T_{{\rm{abs}}}})d\lambda }}}},\end{equation*}

where *I*_BB_(*λ*,*Τ*_abs_) refers to the hemispherical spectral thermal irradiance of a standard blackbody determined by Planck's Law. The thermal radiation loss is dependent on the absorber temperature, leading to a radiation peak in the infrared wavelength range. Therefore, an ideal evaporator is required to possess low absorbance in the thermal emission window except for the efficient light absorption in the solar spectrum illustrated in the above section, which is called the spectrum selective solar absorber. The band edge of selective absorption is determined by the operation temperature. For example, for low-temperature vapor generation such as ISVG in water treatment (*T* <100°C), an absorption band edge around 2.5 μm is sufficient, while for much higher-temperature applications such as Stirling engines or concentrated solar applications, an absorption band edge around 1.5 μm or even shorter would be preferred. Recently, a work by Ni *et al*. was the first to employ a commercial spectrally selective absorber for suppressing radiation loss in ISVG, with a high solar absorbance of 0.93 and a low thermal emissivity of 0.07. The result shows that the radiation loss can be suppressed by about one order of magnitude through selective absorbers [77]. However, there is still huge space to explore for the ideal hybrid absorber–emitters for ISVG due to the inherent difficulty in spectrum tunability of semiconductors as well as fabrication limitations for metallic absorbers [78].

##### The extrinsic strategy to suppress radiation loss.

The origin of the extrinsic strategy is either decreasing the temperature of absorber or recycling the radiated power through structural design. One of the representative routes is to enlarge the effective evaporation area of ISVG. With a large amount of vapor escape taking away pronounced phase-change enthalpy, the surface temperature of absorbers can be greatly reduced. Based on this concept, 3D artificial transpiration devices were proposed to reduce the evaporation temperature of the absorber by effectively increasing its effective evaporation area. As a result, only 8% energy was wasted through convection and radiation loss without insulation container and optical concentration, and an 85% conversion efficiency was achieved [61]. The same strategy has been adopted in other structures such as mushroom-based solar evaporator and 3D absorbers to increase conversion efficiency [36,37,83]. Inspired by this route, researchers have developed an environmentally enhanced solar vapor generators, easily beating the conventional solar evaporation limit through rational designs [30]. In the meantime, another environmentally suppressed radiation route has been demonstrated by Shi *et al.*, in which the emitted thermal energy recycling involved a smart 3D system design [37]. Such a design can enable almost 100% energy conversion efficiency.

#### Suppressing thermal convection loss

Convection is more complicated than thermal conduction and radiation. According to Newton's law of cooling in Equation (5) (valid for temperature difference ∼<50 K), if there is a temperature difference ascribed for thermal convection, it is probably simultaneously accompanied by thermal conductance and radiation loss. Therefore, convection loss is commonly a hybrid thermal transfer effect that is seldom manipulated independently. There are many intrinsic and extrinsic issues that may affect the thermal convection (convection coefficient *h*) between the solar evaporator and ambient fluid: 1) type of fluid (liquid or gas); 2) physical properties of the fluid such as mass density, dynamic viscosity, specific heat capacity, thermal conductivity, etc.; 3) geometric properties of the surface of the evaporator, such as microstructure, surface roughness, surface alignment, etc.; and 4) the flow properties of the fluid (natural or forced convection). It is rather hard to finely manipulate many conditions and almost impossible to decouple the influence from other issues, such as surface wettability [84]. Hence, up to now there has been relatively little research related to thermal convection loss engineering. However, there are still some regularities to follow. One effective strategy is to reduce the affordable space of the fluid [77]. Another is to shorten the temperature gap between absorber and ambient by increasing the effective evaporation area [61].

By combining the above thermal suppression strategies, two ideal ISVG structures with efficient heat management are summarized in Fig. 4d and e. This shows that the heat manipulation has made significant advancement through rational designs of materials and structures regardless of high-temperature or low-temperature evaporation. However, it is still worth exploring more work focusing on material designs for thermal management, especially for radiation and convection heat loss.

### Designs of water/vapor paths

**Figure 5.**
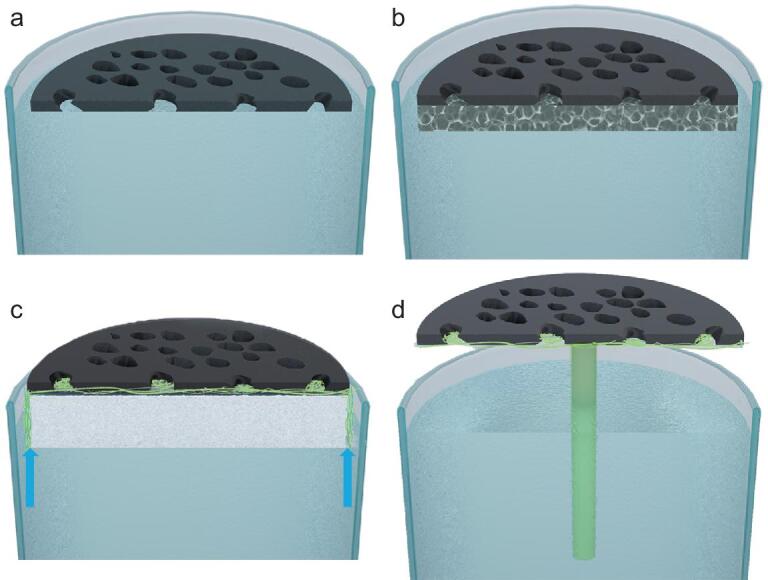
Water path management for ISVG. (a) Self-floating 2D solar absorber with self-built bulk water path. (b) Hybrid self-floating solar absorber with partially confined bulk water path. (c) Thermal insulator-supported 2D water path. (d) 1D water path.

Water and vapor paths are vital for the mass transport process of ISVG, which is coupled with the energy transfer process as illustrated above. An efficient ISVG can only be achieved provided that the water source can directionally flow to the localized heating area and the generated solar vapor can transport far away smoothly. Keeping in mind that mass transport is coupled with the energy transfer, researchers have been trying to optimize the water path all through the development pathways of ISVG (Fig. 1). For self-floating ISVG systems, there is no extra water path since the nanopores or channels behave as a high-density water-path array (bulk-like water path, Fig. 5a) [25,35,47,60,64,78]. In order to optimally offset the trade-off between thermal energy loss and mass transport, a series of ISVG structures and/or systems are demonstrated, primarily featuring decoupled paths of water supply and vapor escape. Figure 5b–d illustrates three representative mass–path-decoupled ISVG systems, which include a double-layer evaporator with 3D water supply (Fig. 5b) [46], 2D water paths with extrinsic thermal insulation floater (Fig. 5c) [14], and 1D water paths of mushroom structures (Fig. 5d) [83], respectively. These smart water/vapor path designs combined with other structural or systematic designs finally increase the one-sun solar vapor efficiency to above 80%.

Based on the above various designs for water paths, we emphasize that an ideal ISVG is far more than a porous structure. For example, the inner walls of water paths are required to be smooth for timely mass transport [67,68]. A certain surface wettability of the structured materials is beneficial for the process as well [22,70]. Most recently, researchers have reported that curved liquid surfaces are ideal provided that the energy barrier and/or extra forces acting on the surface molecules of water are reduced to a certain extent [85–88]. This is so far still an elusive issue that has no clear answers, for which deeper insights for the mass transport process are still required.

Actually, the water flow rate should be self-regulating by the capillary forces, up to a maximum flow rate determined by a maximum capillary pressure. The fastest flow rate for a certain pore structure/capillary design can be suggested, which determines the narrowest water path required for a given evaporation area. Therefore, there is an optimal operation condition in which the water flow rate exactly matches the vapor generation rate, accompanied by minimal thermal energy loss, calling for future research.

Up to now we have summarized a versatile point of using rational designs from photonics to thermal and mass transport, from materials to structures, as well as from intrinsic to extrinsic system levels. Finally, we would like to briefly review the fabrication developments of the reported solar–thermal materials for ISVG. So far, a lot of smart fabrication processes/methods have been developed for membranes, artificial networked structures and so on by researchers, ranging from 3D printing [72], freeze drying [64], thermal annealing [46,56,73,74,83], physical self-assembly [25,47,78], chemical synthesis [13], etc. Taking the thermal annealing treatment as an example, it is an ideal process for generating a highly porous structure, which can be directly used for biomass carbonization. Hu group has developed a novel carbonization process for natural wood, which can produce an inherent double-layer structure for efficient ISVG [73]. Recently, the 3D printing method has drawn widespread attention [72]; this can be programmed and conducted by a benchtop robot, advantageous in large-scale fabrication requirements.

## APPLICATIONS AND OUTLOOK

The conceptual revolution of ISVG has witnessed the revival of conventional solar–thermal-based water technology, which has brought a couple of potential applications as well as breakthroughs in some crucial FOMs.

**Figure 6.**
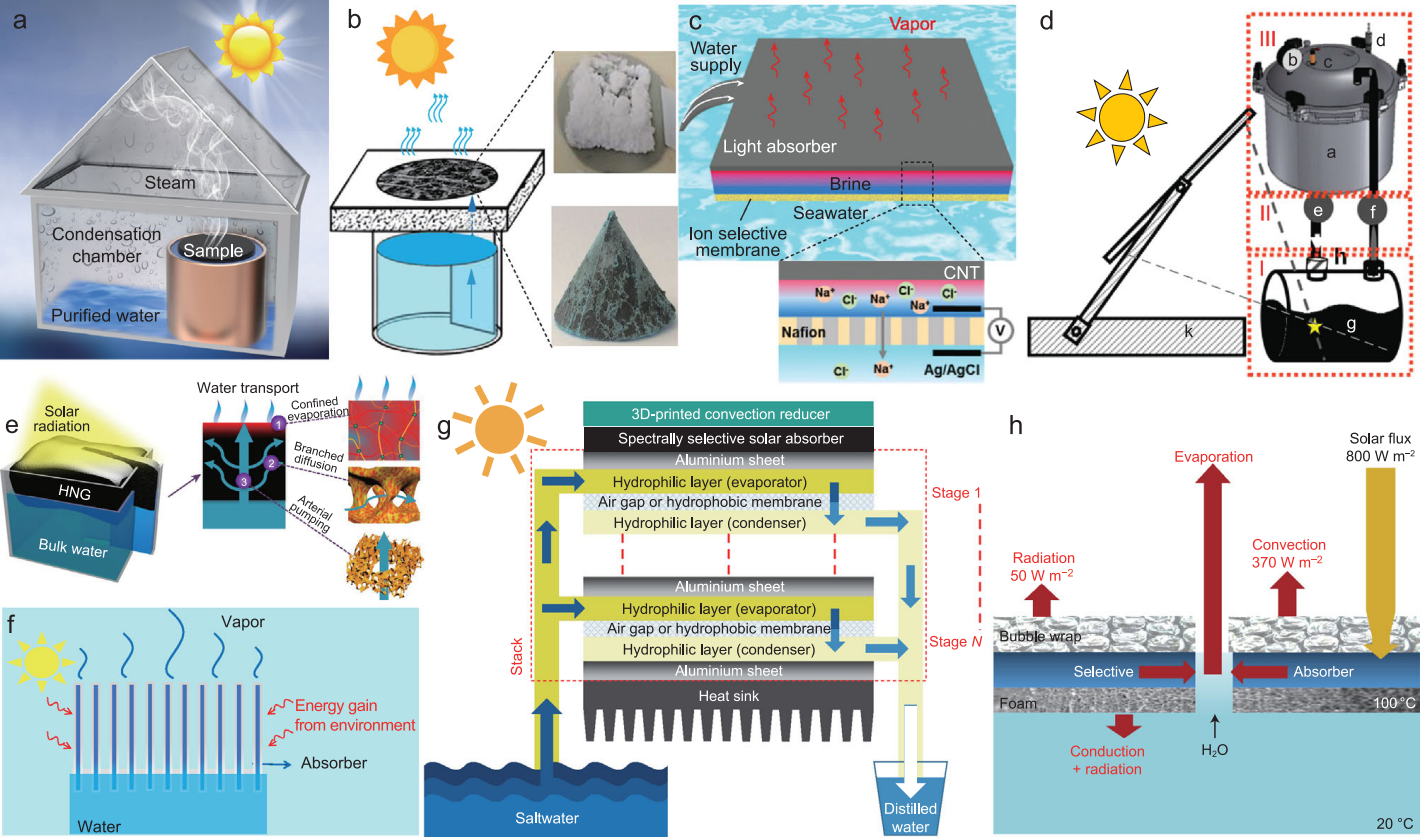
Potential applications of ISVG. (a) ISVG enabled solar desalination [47]. (b) ISVG for wastewater treatment or bay salt [61,63]. (c) ISVG for simultaneous generation of electricity and vapor [89]. (d) ISVG for domestic sterilization [4]. (e) ISVG with hierarchically nanostructured gels [39]. (f) Environment energy enhanced ISVG [36]. (g) Solar distillation design with systematic latent heat recovery [94]. (h) ISVG for vapor under one sun via thermal concentration [77].

Figure 6a–d shows four kinds of conceptual applications enabled by ISVG. Benefiting from the enhanced evaporation rate of ISVG and unique advantages of thermal-based desalination, the passive solar–thermal desalination (Fig. 6a) will be revived for decentralized water purification technologies. The solar evaporation enabled water treatment featured by zero-liquid discharge would be the emerging direction for high-concentration brine treatment, which may be one of the promising point-of-use technologies for interfacial solar evaporation.

Also, due to the enhancement solar vapor evaporation rate as well as the robust mechanical stability of the solar evaporator, ISVG can be an alternative candidate for wastewater treatment with minimal sewage discharge and/or valuable chemical recyclables (such as salts and noble metals; see Fig. 6b). A simple combination of ISVG with a power generation system (see Fig. 6c) [89] or solar fuel system [90] shows great promise for urgent survival needs in special areas with both water and energy shortages. Apart from the evaporation-rate-inspired applications, the high-temperature generation in shortened response time for ISVG can shed light on advanced solar sterilization applications [4,76] for off-grid and/or urgent medical disinfection (Fig. 6d). These promising applications could greatly improve the living quality of human beings and also provide powerful solutions for the inextricably linked dilemma of the water–energy nexus.

It is worth noting that, to essentially convert these promising conceptual applications to large-scale or commercial utilizations, there is still a long way to go. Actually, most bottlenecks of the ISVG-enhanced applications exist in the absolute promotion of the FOMs. For example, the absolute solar vapor generation rate will finally determine whether the solar desalination and wastewater treatment can be accepted by the market, while the vapor temperature as well as the response time will finally determine whether human beings will adopt the ISVG-based sterilization, etc. It is exciting that most recently several milestones have been reached, with new concepts and prospects in the field of ISVG.

We take advanced solar water treatment, the most intensively investigated application by ISVG, as an example. The solar vapor generation efficiency has been greatly improved based on the latest progress, reaching >85% under one sun illumination. However, the absolute vapor production rate under one sun is still not that attractive. Compared to the filtration-based technology in seawater desalination or adsorption-based technology for wastewater treatment, clean water production by ISVG-based solar water treatment is still less productive. Even for portable water purification devices, the water production rate approaching the theoretical limit (1.47 L/m^2^/h) under one sun is still limited. In March 2018, Zhao *et al.* reported exceptionally high solar vaporization rates up to 3.2 L/m^2^/h under natural sunlight with a hierarchically nanostructured gel [39] (Fig. 6e) (comparable to mainstream membrane distillation and even better than most membrane filtration methods under similar salt rejection rates). In addition, the overwhelming evaporation rate breaking through the traditional photothermal efficiency limit also indicates that the phase-change enthalpy of water might be effectively reduced, which is related to hierarchical pathways of water management, especially via the key components of molecular meshes, as claimed by the authors, inspiring both fundamental and applicable research interest in the future. Apart from the fundamental strategy to break through the upper limit of efficiency, very recently, an environmental energy enhanced ISVG has been proposed by Li *et al.*, in which a reversed temperature difference is generated and an evaporation rate of 1.78 L/m^2^/day under one sun is achieved (Fig. 6f) [36]. In addition, there are also successful hybrid-system breakthroughs for higher production rates of water purification, such as solar membrane distillation as well as various phase-change enthalpy recycle strategies [91–93]. The multiple-stage recovery shown in Fig. 6g provides another promising strategy for more water production, which can be achieved via advanced solar membrane distillation combined with multi-stage enthalpy recycle processes [94]. Such a system enjoys advantages of both thermal-based desalination and membrane-based filtration processes. Therefore, they experimentally demonstrated a large-scale prototype that produces purified water at a rate of one order of magnitude higher than the conventional large-scale ISVG systems.

On the other pole of ISVG development that aims at the applications of high-temperature solar vapor, such as solar sterilization and solar cooking as well as solar vapor engines and so on, researchers have also achieved some milestones in high-temperature vapor generation. Unlike the conventional high-temperature strategies that more or less rely on high optical concentration [76,95,96], recently Ni *et al*. have provided a novel strategy known as thermal concentration, as shown in Fig. 6h. It is demonstrated that such a thermal concentration strategy can generate water vapor with elevated vapor temperature by enlarging the ratio of the solar absorption area to the evaporation area in combination with optimized thermal insulation design [77]. Approximately 100°C solar vapor under ambient pressure with a thermal concentration factor of 200 was achieved. Most importantly, all the employed materials and accessories are commercially available at low cost; therefore the thermal concentration enabled high-temperature ISVG can be ∼20 times cheaper than processes involving optical concentration.

From an outlook perspective, we would like to point out that despite the amazing achievements in both advanced solar–thermal materials and state-of-the-art applications in the past few years, there are still significant barriers and a number of challenges, which call for more effort in future research.

- In order to further boost the FOMs of ISVG systems, more effort should be devoted to original innovations and physical understanding aiming at breaking through the conventional boundary conditions. Both theoretical and experimental explorations on small scales, within high spatial resolution and/or ultrafast timescales are highly desirable to reveal the thermodynamic and kinetic processes. From that perspective, *in situ* techniques as well as dynamic theoretical explorations are highly desirable in this field. For example, the water evaporation dynamics in microstructured confined space is of significant importance for further investigations.
- In addition, deeper insights are desirable into the understanding of interfacial resistance and hydrophility manipulation, especially for extensively investigated hybrid solar–thermal materials, which may be beneficial for more directional thermal transport for ISVG.
- To broaden the applicable circumstances of ISVG-based seawater desalination, higher evaporation rates combined with comparable water condensation strategies are urgent. Various heat-loss recycling system designs combined with integrated optical and thermal designs are recommended.
- To generate high-temperature vapor with higher efficiency, weaker optical concentrations and faster response time, new insights are required to offset the balance between the efficiency and temperature of ISVG [77], and to break through the high optical concentration constraint on solar sterilization [97].
- To meet the requirements of large-scale applications [98,99], it is still quite urgent to develop and promote the long-term stability of solar–thermal materials, including thermal stability, chemical stability, mechanical stability, etc. For example, salt-rejecting solar evaporators with high energy efficiency are highly pursued, either by decoupling the solar evaporation area and the salting water at the interface of the hybrid structure [71], by multiple channels for sufficient salt diffusion at the systematic level [100], or revolutionary technologies.
- Apart from the aforementioned conceptual applications, more attention can be paid to other specialized water sources, such as organic wastewater containing highly volatile organic chemicals or microbial contaminants, as well as nearly saturated inorganic ions. Various integrated bifunctional or multifunctional devices with breaking-through performance are highly desirable as well [52,89,90]. With respect to the ISVG-based integrated devices, one of the most important issues is to aim at clear application situations instead of merely staying at the conceptual level for one or two functions, bringing the ISVG towards real applicable technologies.

## Supplementary Material

nwz030_Supplemental_File
