# Supplementary material for: The revival of thermal utilization from the Sun: interfacial solar vapor generation
Source: Natl Sci Rev. 2019 Mar 4;6(3):562–78. doi: 10.1093/nsr/nwz030 (PMC8291486; doi:10.1093/nsr/nwz030)
Supplement: nwz030_Supplemental_File [file nwz030_supplemental_file.docx]

**Supplementary data**

**The revival of thermal utilization from the Sun: interfacial solar vapor generation**

Lin Zhou^1^, Xiuqiang Li^1^, George W. Ni^2^, Shining Zhu^1^ and Jia Zhu^1,*^

^1^National Laboratory of Solid State Microstructures, College of Engineering and Applied Sciences, School of Physics, Key Laboratory of Intelligent Optical Sensing and Manipulation, Ministry of Education, and Collaborative Innovation Center of Advanced Microstructures, Nanjing University, Nanjing 210093, P. R. China.

^2^Department of Mechanical Engineering, Massachusetts Institute of Technology, Cambridge, Massachusetts 02139, USA.

^*^ Email: jiazhu@nju.edu.cn

**Note S1. Setup of ISVG measurements**

The ISVG measurements are based on a real-time solar vapor generation measuring system, as shown in panel **a.** Apart from the test chamber (with floating solar evaporator and water) as well as optical accessories, the four necessary components are solar simulator, electronic balance with computer, thermal power meter and infrared camera or thermal couples, respectively. In order to fully evaluate the ISVG process, it is strongly recommended to stabilize the solar simulator and all the measuring elements before using them. In addition, it is necessary to record all the initial and boundary conditions of the measuring system, for example, the ambient temperature, relative humidity, the optical power fluctuation of the solar simulator during the measurements as well as water quantity and temperature, and to make sure the dark evaporation experiment conducted under the same condition except for the solar irradiance. In order to obtain precise experimental results of mass change, optical power and temperature profiles, there are several crucial issues for careful treatments as illustrated below.

**S1.1 On-site mass change measurement.**

The real-time mass change record is crucial for the calculation of evaporation rate. In order to precisely record the mass change, a high accuracy electronic balance (0.1 - 1 mg) combined with an automatic data collection system is recommended. In addition, proper ambient conditions (air convection, mechanical stability of operation desk) are required to enable a normal operation state of the electronic balance. The mass data as a function of time is recorded, based on which the steady state evaporation rate (or *dm*/*dt*) is subtracted by post-processing (Supporting Information, **Fig. S1b**). Moreover, the response time can also be obtained via post-processing of mass data as a function of time (Supporting Information, **Fig. S1b**).

**S1.2 Optical measurements.**

Two optical parameters crucial for ISVG are the input optical power density *P_solar_* and absorbance of the solar absorber *α_eff_*. To ensure precise measurement, *P_solar_* should be measured by a thermal pile or optical power meter based on thermal effect while *α_eff_* by the integrated-sphere equipped broadband spectrometer (see inset of Supporting Information, **Fig. S1c**). In order to simulate the solar evaporation under natural sunlight, AAA grade solar simulator is recommended, which shows better spectral accordance with the standard solar irradiance of AM 1.5G than at least Xe lamps and other light sources [23]. Supporting Information, **Fig. S1c** shows the spectral difference between AM 1.5G solar spectral irradiance and a Newport solar simulator (94043A). It is clear that distinct discrepancy can be found especially in the range of 300-500 nm and 800-1000 nm. Similar systematic errors (3 - 4% of input power density) can also be found in Xe lamps or other light sources. Therefore, as an important pre-calibration, one must calibrate the input optical power density at the exact working plane (the absorber/air interface) at each time of the experiment, which is crucial for wavelength-dependent absorber or applications.

**S1.3 Thermal measurements.**

Two thermal parameters crucial for ISVG is the temperature of vapor and liquid as well as the thermal conductivity. The temperature of vapor and liquid can be measured by thermocouples widely employed in the early stage for the low cost and convenient operation. However, the unwanted thermal conduction loss and disturbance to the floating system makes it improper for simultaneous records of mass change and temperature. IR-camera is now widely adopted for the indirect contact with vapor or water. In order to obtain accurate temperature data, the IR camera has to calibrate carefully and an ideal angle for photography is needed.

Thermal conductivity of solar absorber or evaporator is usually measured by IR microscope. In order to mimic the real operation condition of ISVG, the target solar absorber for thermal measurement is required in wet state, which indicates that conventional measuring systems for thermal conductivity are improper for ISVG systems. A general setup is shown **Fig. S1d**, in which the target solar absorber (sandwiched between two substrates such as glass slabs or aluminum blocks) thermally contacts with heat source and sink on each side, respectively. During the measurement, the heat sink is maintained at a ﬁxed temperature, while power of the heat source is tuned to generate a set of temperature gradients across the sandwich structure. The longitudinal temperature distribution across the layered structure is monitored by IR camera. The heat flux can then be obtained by measuring temperature gradient for a given referenced material with known thermal conductivity. Once the heat ﬂux is obtained, the thermal conductivity of the target solar absorber materials can then be calculated by the Fourier law with measured temperature gradient in the sandwich structure.

**S1.4 Other extrinsic conditions**.

Apart from the above three parameters, a series of environmental issues should be noted as well for researches so that our peers can obtain justified evaluation and valuable information. These environmental issues include the ambient temperature, water temperature, ambient humidity, sufficient working distance between test chambers and other equipment with high temperature and/or light emission, *etc*.


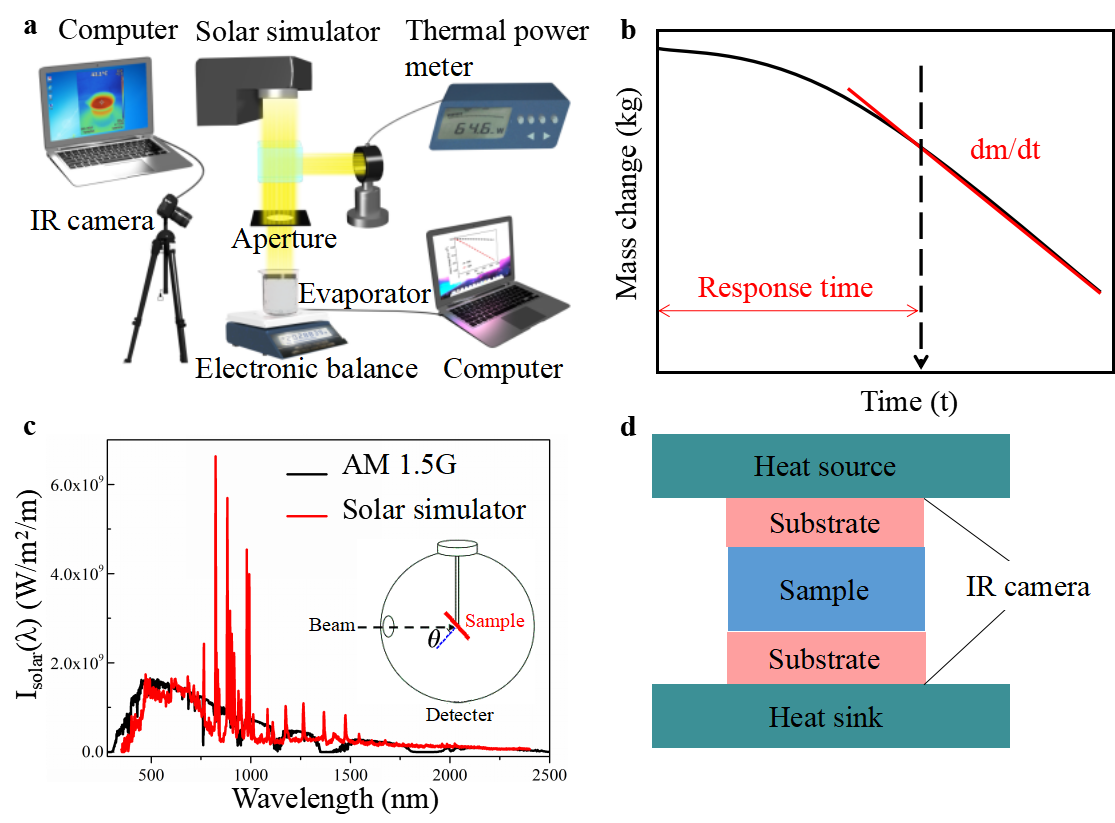


**Figure S1.** Schematic for protocols in characterization of interfacial solar vapor generation. (a) Standard experimental setup for ISVG. (b) Identification of steady state solar evaporation rate. (c) Standard solar absorption measurement configuration. (d) schematic of thermal conductivity measurement.
